# Evaluation of quercetin as a potential β-lactamase CTX-M-15 inhibitor via the molecular docking, dynamics simulations, and MMGBSA

**DOI:** 10.3906/kim-2011-52

**Published:** 2021-08-27

**Authors:** Emrah SARIYER*, Ayşegül SARAL

**Affiliations:** 1 Artvin Çoruh University, Vocational School of Health Services, Medical Laboratory Techniques, Artvin Turkey; 2 Artvin Coruh University, Faculty of Health Sciences, Department of Nutrition and Dietetics, Artvin Turkey

**Keywords:** β-lactamases, antibiotic resistance, quercetin, molecular docking, molecular dynamics simulations

## Abstract

Antimicrobial resistance (AMR) threatens millions of people around the world and has been declared a global risk by the World Economic Forum. One of the important AMR mechanisms in Enterobacteriaceae is the production of extended-spectrum β-lactamases. The most common ESBL, CTX-M β-lactamases, is spread to the world by CTX-M-15 and CTX-M-14. Sulbactam, clavulanic acid, and tazobactam are first-generation β-lactamase inhibitors and avibactam is a new non-β-lactam β-lactamase inhibitor. We studied that avibactam, sulbactam, clavulanic acid, tazobactam, and quercetin natural flavonoids were docked to target protein CTXM-15. Subsequently, the complexes were simulated using the molecular dynamics simulations method during 100 ns for determining the final binding positions of ligands. Clavulanic acid left CTX-M-15 and other ligands remained in the binding site after the simulation. The estimated binding energies were calculated during 100 ns simulation by the MMGBSA-MMPBSA method. The estimated free binding energies of avibactam, sulbactam, quercetin, tazobactam, and clavulanic acid were sorted as –33.61 kcal/mol, –16.04 kcal/mol, –14 kcal/mol, –12.68 kcal/mol, and –2.95 kcal/mol. As a result of both final binding positions and free binding energy calculations, Quercetin may be evaluated an alternative candidate and a more potent β-lactamases inhibitor for new antimicrobial combinations to CTX-M-15. The results obtained in silico studies are predicted to be a preliminary study for in vitro studies for quercetin and similar bioactive natural compounds. These studies are notable for the discovery of natural compounds that can be used in the treatment of infections caused by β-lactamase-producing pathogens.

## 1. Introduction

Antimicrobial resistance (AMR) is one of the most important public health problems worldwide which threatens the prevention and treatment of infections caused by microorganisms [1]. The production of extended-spectrum β-lactamases (ESBL) is one of the most important AMR mechanisms in *Enterobacteriaceae* [2].

CTX-M enzymes classified in class A ESBLs have strong cefotaxime hydrolyzing activity [3]. These enzymes were divided into 5 subgroups. CTX-M-1, CTX-M-2, CTX-M-8, CTX-M-9, and CTX-M-25 there are more than 124 variants [3]. CTX-M-15 is the most common ESBL in Gram-negative bacteria and has been reported in Poland, India, Britain, Bulgaria, Romania, and Turkey. CTX-M-15 has a broad spectrum of β-lactamase activity on various antibiotics and shows high activity against cephalosporins, including cefotaxime and ceftazidime. The rapid spread of this enzyme in pathogens that cause hospital and community-acquired infections affects treatment options in hospital settings [4]. 

Tazobactam, clavulanic acid, and sulbactam are the first generation β-lactamase inhibitors and received FDA approval between 1984 and 1993 [5]. The activity spectrum of clavulanic acid includes most class A β-lactamases containing ESBLs and to lesser extent serine carbapenemases. Sulbactam and tazobactam had a similar spectrum of activity with clavulanic acid [6]. β-lactamase inhibitors prevent the breakdown of β-lactam antibiotics by β-lactamases and they are used in combination with β-lactam compounds. β-lactam/β-lactamase inhibitor combinations allow restoring the efficacy of β-lactams [7]. The β-lactam ring forms the core structure of sulbactam, clavulanic acid, and tazobactam. Various mechanisms have been found in bacteria that confer resistance to these inhibitors. These are hydrolysis of the core structure of the inhibitor, mutation in porin channels, and overexpression of β-lactamases [8]. These three inhibitors inactivate CTX-M-15 but at the same time, these inhibitors tend to be hydrolyzed by CTX-M-15 [9]. Since strains containing CTX-M-15 can be developed resistance to these inhibitors, studies should be carried out for the discovery of new inhibitors with non-β-lactam core structure. New natural or synthetic inhibitors that do not have this β-lactam core structure will not be affected by porin channel mutations that prevent β-lactams from reaching penicillin binding proteins (PBPs) and will not be hydrolyzed by β-lactamases. Also, it will take a long time for bacteria to develop new resistance mechanisms against these new inhibitors [8].

Avibactam, a member of the diazabicyclooctane family, is a novel synthetic non-β-lactam/β-lactamase inhibitor. It shows activity against class A, class C, and some class D enzymes [10]. Avibactam inhibits CTX-M type β-lactamases, like first-generation β-lactamase inhibitors [11]. In addition to the discovery of new synthetic β-lactamase inhibitors that do not have β-lactam core structures like avibactam, it is possible to find inhibitor candidates with this feature among natural bioactive compounds.

Bioactive compounds (phytochemicals) include tannins, alkaloids, carbohydrates and glycosides, terpenoids, steroids, flavonoids, and coumarins [12]. Quercetin, a flavonoid, has β-lactamase inhibiting activity and does not contain a β-lactam ring in its chemical structure [13]. Quercetin has been found to interact with the active site of TEM-1 and SHV-1 and has higher binding energy than clavulanic acid [14].

We studied the potential of Quercetin, which is a natural compound and does not contain a β-lactam ring for a β-lactamase inhibitor. Since CTX-M-15 is able to hydrolyze β-lactam core structures, it has been motivated to discover new inhibitors having a non-β-lactam ring. For this purpose, avibactam, sulbactam, clavulanic acid, tazobactam, and quercetin were docked to CTX-M-15 by molecular docking methods and were simulated by MD methods during 100 ns to determine their binding positions. Besides, the binding energy of quercetin to CTX-M-15 was determined by MMGBA calculations. At the same time, the binding energies of the first generation β-lactamase inhibitors (clavulanic acid, sulbactam, and Tazobactam) and Avibactam to CTX-M-15 will be calculated using the MMGBSA method. The outputs will allow the evaluation of quercetin which does not contain β-lactam ring and reveal the inhibitory potential of this phytochemical. It is predicted that candidate inhibitors identified by the data in this study can be used for further in vitro and in vivo studies. 

## 2. Materials and methods

### 2.1. Materials

3D structures of avibactam (PubChem CID: 9835049), sulbactam (PubChem CID: 130313), clavulanic acid (PubChem CID: 5280980), tazobactam (PubChem CID: 123630), and quercetin (PubChem CID: 5280343) were obtained as SDF format from PubChem chemical database [15]. 2D structures of avibactam, sulbactam, clavulanic acid, tazobactam and quercetin were illustrated in Table. But, the 3D crystal structure of target protein CTX-M-15 (PDB ID:4S2I) was now available at RCSB Protein Data Bank. The structure from the database has a resolution of 1.8 Å and the structure was defined by the X-ray diffraction method [16].

**Table:** T2D structures and names of ligands [17].

Avibactam[(2S,5R)-2-carbamoyl-7-oxo-1,6-diazabicyclo[3.2.1]octan-6-yl] hydrogen sulfate 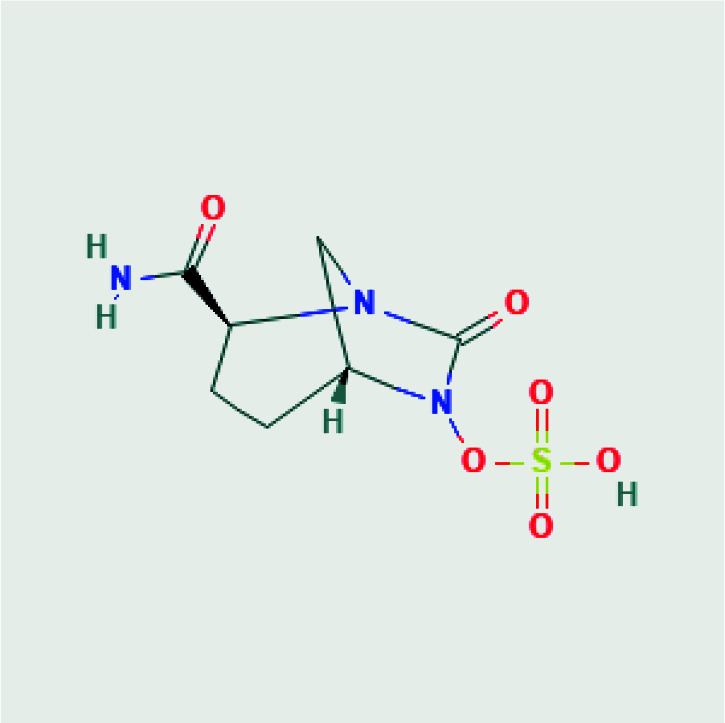	Sulbactam(2S,5R)-3,3-dimethyl-4,4,7-trioxo-4λ6-thia-1-azabicyclo[3.2.0]heptane-2-carboxylic acid 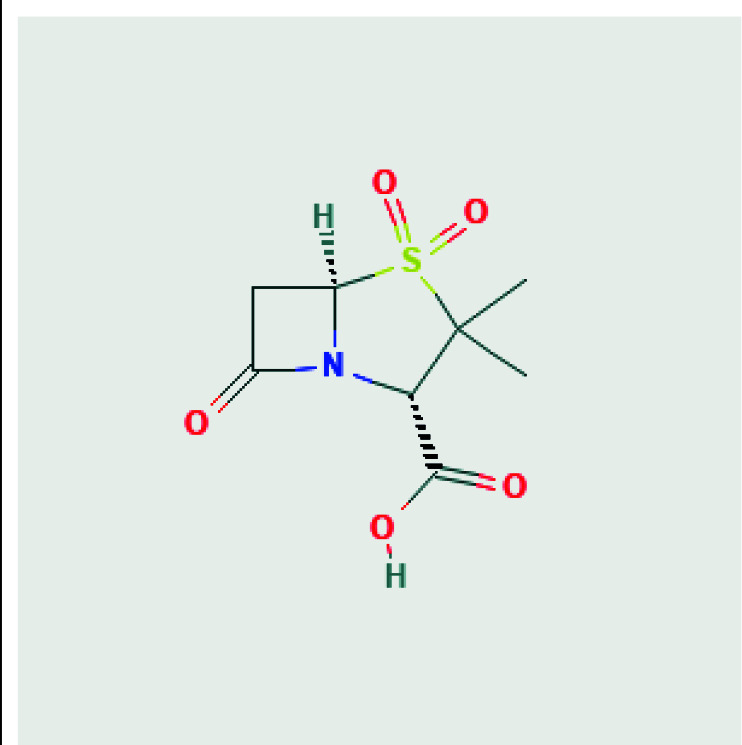
Tazobactam(2S,3S,5R)-3-methyl-4,4,7-trioxo-3-(triazol-1-ylmethyl)-4λ6-thia-1-azabicyclo[3.2.0]heptane-2-carboxylic acid 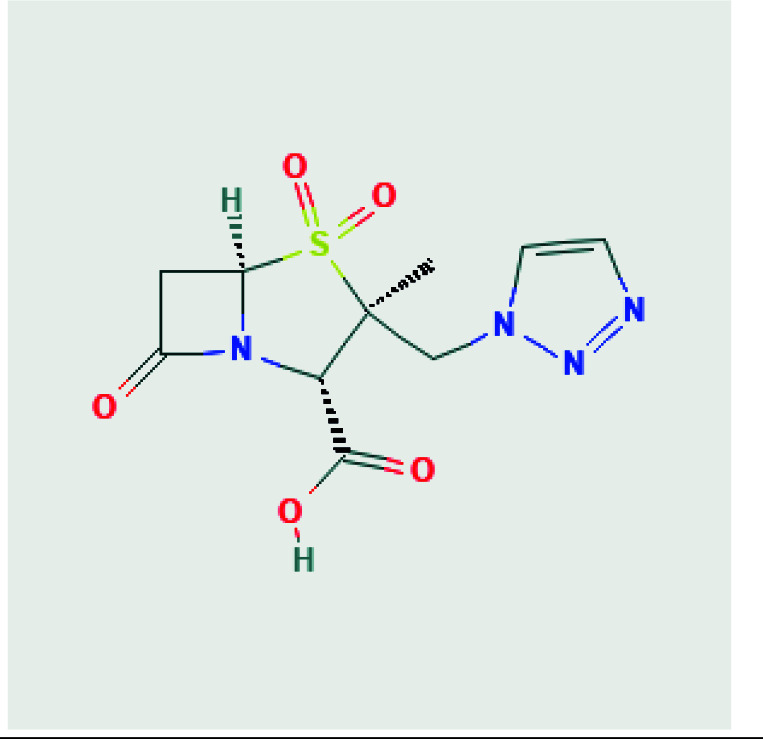	Clavulanic-acid,(2R,3Z,5R)-3-(2- hydroxyethylidene)-7-oxo-4-oxa-1-azabicyclo[3.2.0]heptane-2-carboxylic acid 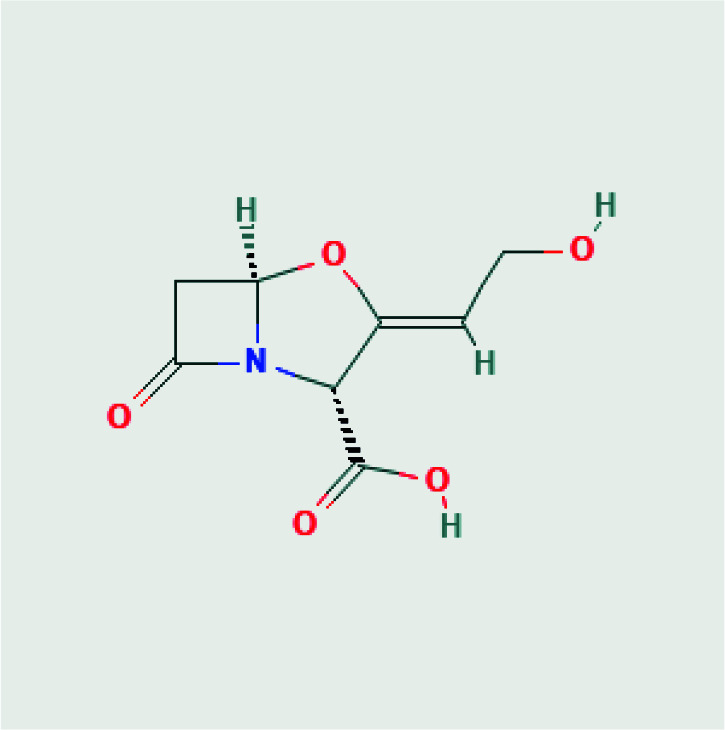
Quercetin-(3,4-dihydroxyphenyl)-3,5,7-trihydroxychromen-4-one 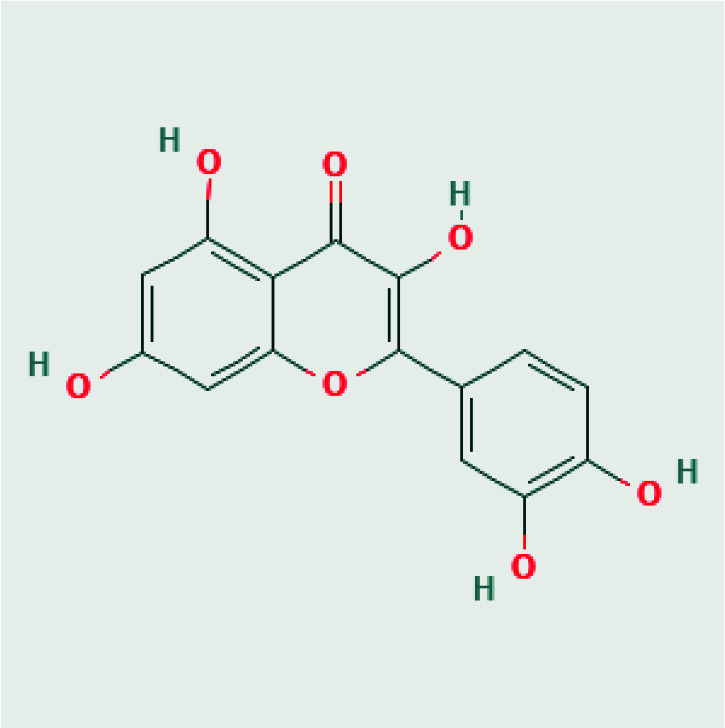	

### 2.2. Molecular docking of ligands

The catalytic site of the enzyme was targeted using AutoDock4.2 [18]. Firstly, the coordinates types of the ligands and CTX-M-15 were converted to the pdbqt format Autodock4.2 could read. A grid map was created using the AutoGrid tool and wrote as a grid parameter file. Avibactam, sulbactam, clavulanic acid, tazobactam, and quercetin natural flavonoid were docked to target protein ctx-m-15 and further to compare the minimum binding energy of these ligands. AutoDock searches the binding position using a genetic algorithm and wrote the docking parameter file using Lamarckian [19]. Ten different positions were identified and sorted according to their binding energies by preparing parameter files for molecular docking.

### 2.3. Molecular dynamics simulations of ligand-enzyme complexes

The charge parameters of avibactam, sulbactam, clavulanic acid, tazobactam, and quercetin were derived using AM1-BCC atomic charge [20] and GAFF (general AMBER force field) parameters [21] via Antechamber [22]. Topology and coordinate files of complexes were created using AMBER ff14SB [23] by tLeap. The systems were solvated with TIP3PBOX [24] water molecules in an octahedral box size 10 Å and then were neutralized by adding Cl^–^ and Na^+^ ions. Complexes are minimized energetically to remove bad contacts and clashes with these input files prepared for simulation. The energy minimization was carried out with 10000 steps of Steepest Descent [25] followed by 90,000 steps of conjugate gradient algorithms [26]. When energy minimization is first started with the conjugate gradient algorithm, systems designed using type 3 water generally fail. Therefore, the steepest descent algorithm is first used to loosen collisions and bad interactions between atoms for finding a local minimum. Following, conjugate gradient algorithm which is faster than the steepest descent algorithm finds the unique global minimum [27].

After energy minimization for 10,000 steps followed by heating and an initial equilibration for 200 ps. The systems were heated from 10 K to 300 K with constant volume and pressure using Langevin thermostat [28] with a collision frequency of γ = 10.0 ps^–1^. MD simulations were performed for 100 ns with a time step of 2 fs and the SHAKE algorithm [29] was applied to restraint all atoms during the dynamics run. The temperature was controlled using Berendsen the weak-coupling algorithm [30] during molecular dynamics. The deviations between the positions were evaluated using the root mean square deviation (RMSD) and B-factor plot subtracted using CPPTRAJ [31] from MD simulation trajectory during 100 ns for complexes. 

### 2.4. MM/PBSA and MM/GBSA calculations of the binding free energy for the complexes

An estimate of the binding free energy of avibactam, sulbactam, clavulanic acid, tazobactam, and quercetin were calculated using MM/GBSA and MM/PBSA methods in AMBER14. These methods are post-simulation trajectory analysis methods that approximately calculate the free energy changes of ligands in bound and free states. MM/GBSA binding energy was calculated using the modified GB model [32] in 0.1 M salt concentration from trajectory files read every 50 frames. MM/PBSA were also calculated without nonpolar solvation free energy using atomic radii from the topology file during 100 ns MD simulation.

## 3. Results and discussion

### 3.1. Molecular docking of ligands to CTX-M-15

**Figure 1:**
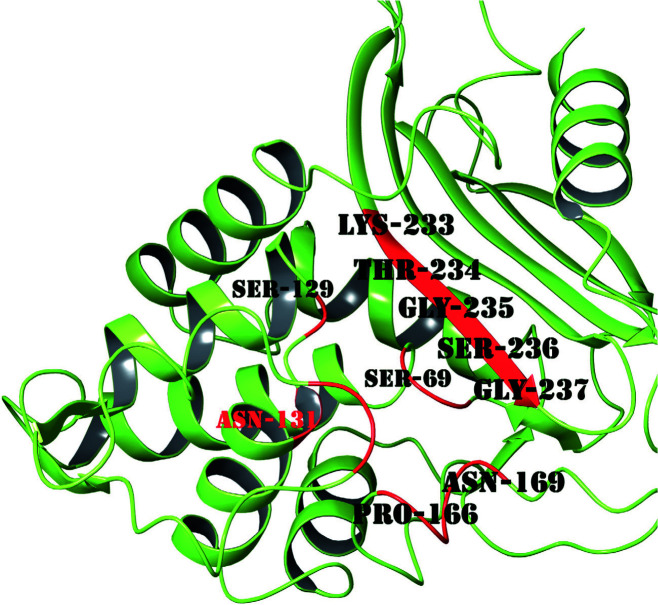
Representation of the putative binding site of CTX-M-15.

Molecular docking methods are performed in two steps as the appropriate binding position of ligands and binding energy calculation. These scores are used to evaluate the thermodynamical equilibrium and formation of noncovalent interactions, salt bridges, and hydrogen bonds of protein-ligand conformations [33]. The putative active site of the CTX-M-15 consists of CYS68, SER69, ASN103, TYR104, SER129, ASN131, PRO166, ASN169, LYS233, THR234, GLY235, SER236, and GLY237 residues (Figure 1) [9]. Avibactam, sulbactam, clavulanic acid, tazobactam, and quercetin were docked to the CTX-M-15 by targeting this region. Avibactam, sulbactam, clavulanic acid, tazobactam β-lactamases inhibitors, and quercetin natural flavonoids were docked to target protein CTX-M-15 using molecular docking methods and further to compare the minimum binding energy of these ligands. Autodock4.2 program determines binding modes and energy between the receptors and the ligands. The binding energy obtained using the program is used to predict and define the efficacy of the binding. The estimated binding energy of avibactam, tazobactam, clavulanic acid, and sulbactam was calculated as –5.76 kcal/mol, 5.39 kcal/mol, –4.98 kcal/mol, and –4.76 kcal/mol, respectively. Besides, the estimated binding energy of quercetin natural flavonoid was calculated as –6.69 kcal/mol using the molecular docking method. According to these results, the binding affinity of Quercetin, which has 3 rings and 5 hydroxyl groups in its structure, was higher than other β-lactamase inhibitors based on docking calculations.

The interactions of the ligands docked to the binding site were analyzed with a 2-dimensional diagram. Avibactam is located in the cavity with polar residues and the THR215 hydrogen bond with the sulfate group, while the azanide group is located in the active site cavity of the enzyme. Sulbactam also involves 3 hydroxyl groups and it seems to form a hydrogen bond with SER236 residue. As it has a smaller structure, it interacts less in the polar pocket. Since it contains 3 hydroxyl groups in its clavulanic acid structure, it formed two hydrogen bonds with its polar residual SER236 and the structure was placed in a completely polar pocket. Tazobactam was settled in the polar and hydrophobic cavity and does not form a hydrogen bond. However, it formed a pi-pi stacking, which is a noncovalent bond, with the TYR104 residue owing to aromatic rings in its structure. Quercetin is also composed of 5 hydroxyl groups and 3 aromatic rings and hydroxyl groups formed hydrogen bonds with polar residues SER219 and ASN131. Quercetin completely overlapped with beta-layer structures in the binding site of the enzyme. It is located in the cavity, a large polar pocket, and appears to form a tight structure. Also, it is revealed that due to the negatively charged and hydrophobic residues in the cavity, the binding affinity increases.

### 3.2. Molecular dynamics simulations of CTX-M-15-ligand complexes

**Figure 2:**
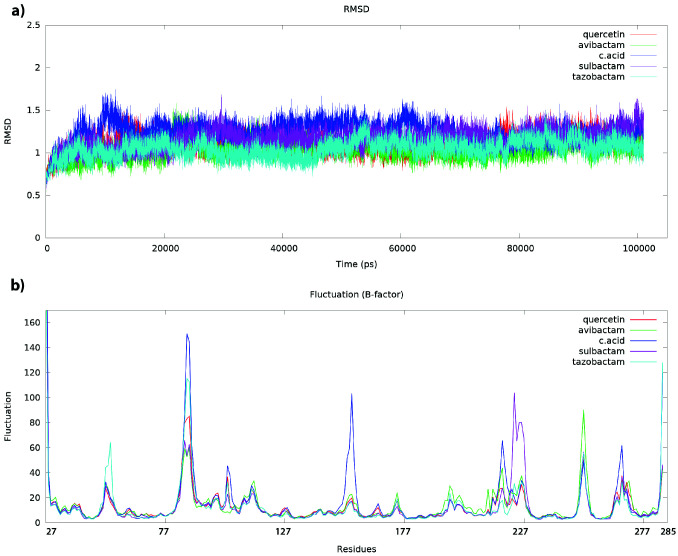
a) Root-mean-square deviations (RMSD) of all Cα backbone carbon atoms for CTX-M-15-ligand complexes b) Per residue atomic fluctuations (β-factors) of the Cα backbone carbon atoms for CTX-M-15-ligand complexes

The complexes of avibactam, sulbactam, clavulanic acid, tazobactam, and quercetin were docked to target protein CTX-M-15 were simulated for 100 ns by molecular dynamics methods to reveal information about whether the ligands docked at the target molecule remained stable at the initial binding position. Structure-function relationships of protein-ligand complexes or naked proteins can be defined by MD simulations methods. The scores on which this mobility is analyzed are usually root mean square deviation (RMSD) and root mean square fluctuations (RMSF) [34]. The effects of ligands on the secondary or primary structures of the CTX-M-15 were analyzed considering the atomic fluctuation (-factor) of each residue Cα backbone and root mean square deviation (RMSD). The average RMSD’s of avibactam, sulbactam, clavulanic acid, tazobactam, and quercetin with CTX-M-15 was calculated as 1.04, 1.16, 1.23, 1.05, and 1.10 during 100 ns, respectively (Figure 2a). The deviation of all complexes began to stabilize after about 20 ns and the RMSD lines became a plateau. Parallel five MD simulations were sufficient to revealed conclusions from whether the ligands are bound to the enzyme or not and which position of the ligands is. Also, the fluctuations of residues backbone were analyzed with the -factor graph calculated from RMSF during the simulation. 

**Figure 3:**
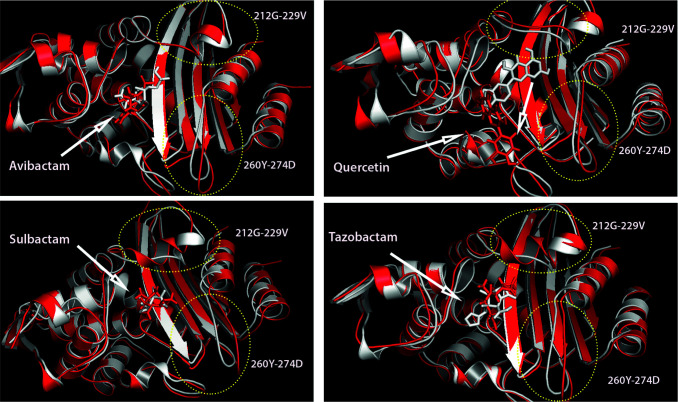
Superimpositions of initial and final conformations of CTX-M-15-ligand complexes. The representation in the ellipse is a higher fluctuated structure. The initial structure was shown in white and the final structure was shown in red.

The higher fluctuation that came to the fore in this graph was the regions between 80–90, 212–229, 248–256, and 261–274 residues (Figure 2b). The structures between residues 80–90 and 248–256 are primary structures and are outside the binding site. Atomic fluctuations were expected to be higher during simulation since they do not have any secondary conformation (Figure 3). Likely, the dynamics of these regions will not affect the function of the protein. However, the regions between 212–229 and 261–274 residues are in the putative ligand-binding site. The fluctuation of these regions is directly proportional to the properties of the ligands attached to the cavity. 

The average RMSD of clavulanic acid was higher than avibactam, sulbactam, tazobactam, and quercetin during the simulation. In snapshots taken at different times, it appears that the ligand was moved away from the cavity to which it is bound after 10 ns and this instability increases the deviation of the system. Avibactam and tazobactam, which have the lowest average RMSD during the simulation, are stable in the position they are bound to the first position (Figure 3). On the other hand, sulbactam and quercetin changed in interactions as the position changed in the hydrophobic cavity they were bound to, and therefore the average RMSD increased (Figure 3). In particular, the fluctuation of the structure between 212–229 residues in the binding cavity, where Sulbactam is supposed to interact, has been calculated to be higher than other complexes. When the 3-dimensional conformation of Sulbactam-CTX-M-15 complex was analyzed, the primary structures in this region have been partially transformed into an α-helix structure. Therefore, the fluctuation of this region during simulation caused higher levels than other complexes.

**Figure 4:**
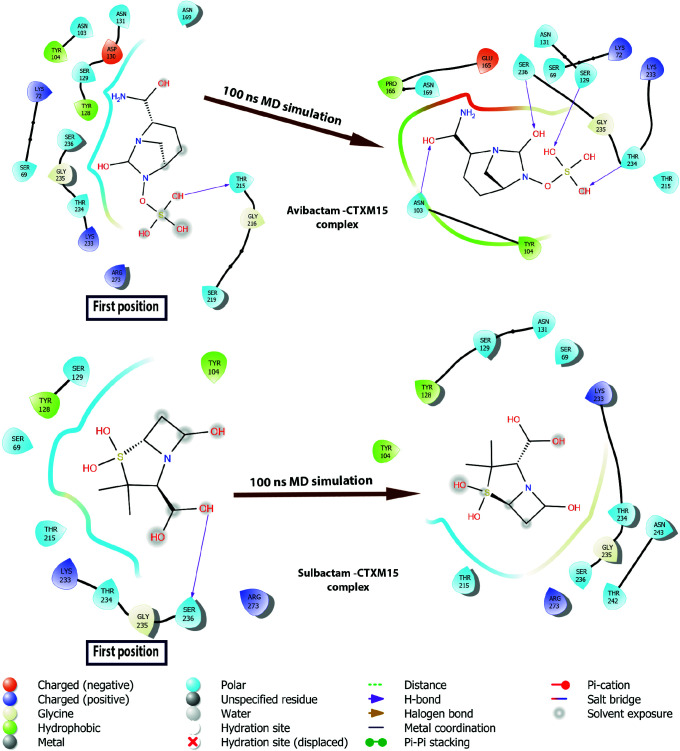
Two dimensional diagrams of interactions of CTX-M-15-Ligands. Before and after simulations of avibactam-CTX-M-15 and sulbactam-CTX-M-15 complexes were shown comparatively in the diagram.

The initial and 100 ns MD post-simulation positions to which the ligands are bound to the CTX-M-15 were compared. These two-dimensional diagrams show interactions between proteins and ligands. Schrödinger Maestro [35] was used in 2D ligand-protein interaction (Figure 4). 2D ligand interaction diagram of Avibactam-CTX-M-15 revealed that avibactam interacted with THR215 in the hydrophobic cavity in the CTX-M-15 using the molecular docking method. But, avibactam formed a hydrogen bond with ASN103, SER129, THR234, and SER236 residues after 100 ns MD simulation and interacted tightly with the enzyme (Figure 4). Sulbactam, which is a smaller compound compared to Avibactam, has lost its hydrogen bond with SER236 since it flips at the end of the simulation in its initial position. However, sulbactam remained stable in the hydrophobic cavity to which it was bound (Figure 4).

**Figure 5:**
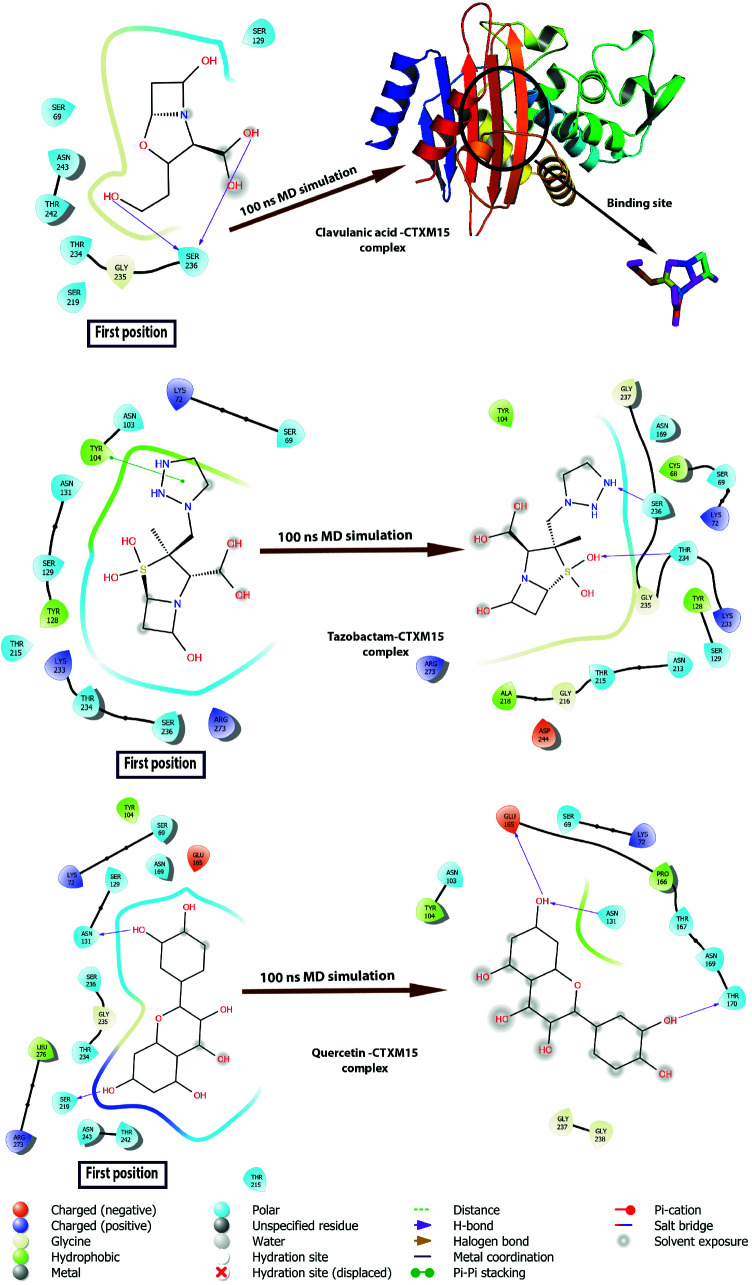
Two dimensional diagrams of interactions of CTX-M-15-ligands. Before and after simulations of clavulanic acid-CTX-M-15, tazobactam-CTX-M-15, and quercetin-CTX-M-15 complexes were shown comparatively in the diagram.

Clavulanic acid contains a β-lactam ring and 3 hydroxyl groups in the structure. It was placed in the hydrophobic cavity by molecular docking methods and formed two hydrogen bonds with SER236. However, SER236 is located in three beta-sheet structures in the binding site and is in a more stable structure compared to loops between 212–229 and 261–274 residues (Figure 1). Since the ligand is smaller than the size of the pocket it was bound to, it lost its interaction with SER236 during the simulation and moved away from the binding cavity (Figure 5). Tazobactam a structure similar to that of Sulbactam contains an aromatic ring attached to the β-lactam ring and four hydroxyl groups. The aromatic group of tazobactam, located in the hydrophobic cavity of the enzyme, pi-pi stacking interacts with TYR104, which has a 6-carbon aromatic ring in the reagent group. Throughout the simulation, Tazobactam was replaced in the hydrophobic cavity to which it was bound, forming hydrogen bonds with THR234 and SER236 residues in three beta-sheet structures in the binding site (Figure 5). 

Quercetin has a larger structure that has 5 hydroxyl groups and 3 aromatic rings compared to 4 compounds known as other β-lactamase inhibitors avibactam, sulbactam, clavulanic acid, and tazobactam. Quercetin, a polyphenolic flavonoid, has potential antibacterial agents, antioxidant properties and reacts with free radicals to give proton. The 2,3-alkene, and the 3- and 5-hydroxyl groups interact with the quercetin beta ring and can give electrons with functional groups [36]. The 3 aromatic rings perfectly overlapped the binding site of the CTX-M-15 with the molecular docking method and interacted with ASN131 and SER219 (Figure 5). After MD simulation, quercetin formed a hydrogen bonding with negatively charged residues ASN131, hydrophobic residues GLU165, and THR170. Quercetin was also moved away from the 3 beta-sheet structures in the binding site. 

### 3.3. MM/PBSA and MM/GBSA calculations of the binding free energy for the complexes

**Figure 6:**
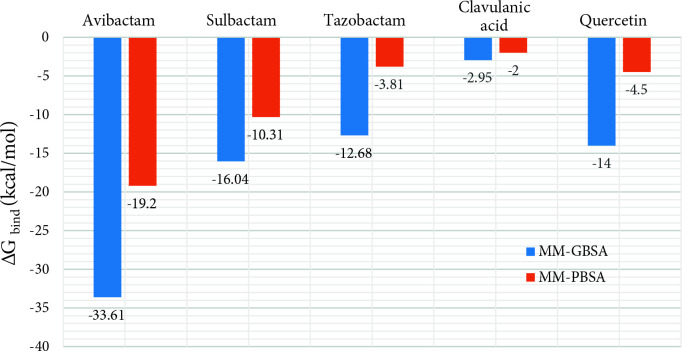
The free binding energy of avibactam, sulbactam, quercetin, tazobactam, and clavulanic acid in CTXM-15.

Poisson–Boltzmann or generalized Born and surface area continuum solvation (MM/PBSA and MM/GBSA) methods calculate the estimated free binding energy of binding small compounds or peptides to biological macromolecules. These calculations are based on molecular dynamic simulation and post-simulation analysis [37]. The MM-GBSA and MM/PBSA methods approximately calculate the energy difference when molecules are bound to the receptor or not [38]. It was aimed to calculate the estimated free binding energy of quercetin which may be a more potent β-lactamase inhibitor for new antimicrobial combinations to CTX-M-15. Binding energies of clavulanic acid, sulbactam, tazobactam, and avibactam to CTX-M-15 were also calculated using the MMGBSA method. MMGBSA calculates binding energy between receptor and ligand using molecular dynamic calculations and implicit solvent methods (ΔGbind = ΔEgas(ΔVDWgas + ΔEELgas) + ΔEsolv(ΔEGBsolv + ΔSURFsolv) kcal/mol). 2000 snapshots were extracted from 100 ns simulation trajectory at 50 ps intervals and 0.1 M salt concentration and a Born implicit solvent model was used in the calculation for each system. The free binding energies of avibactam, sulbactam, quercetin, tazobactam, and clavulanic acid were calculated as –33.61 kcal/mol, –16.04 kcal/mol, –14 kcal/mol, –12.68 kcal/mol, and –2.95 kcal/mol from low to high (Figure 6). Quercetin has a similar or better binding affinity than tazobactam, sulbactam, and clavulanic acid inhibitors having the β-lactam structure according to these results.

## 4. Conclusion

This study aims to find a candidate inhibitor as an alternative to the currently used β-lactamase inhibitors. Molecular docking, molecular dynamic simulation, and MMGBSA energy calculation studies were performed to reveal the potential of quercetin as an inhibitor, which is a natural compound that does not contain a β-lactam ring in its structure. Molecular docking-based binding affinity was sorted as quercetin > avibactam > tazobactam > clavulanic acid > sulbactam from high to low. It revealed from these results that quercetin showed better binding affinity than other known inhibitors. However, the results found the binding positions and energies of the ligands with the molecular docking method only help us make an inference. For this reason, the complexes were simulated by MD methods for 100 ns for the final binding positions of the ligands in the CTX-M-15 and MMGBSA binding energy calculations. As a result of MD simulation study, it was seen that clavulanic acid was moved away from the cavity to which it was bound but other ligands remain stable in the cavity. In particular, Avibactam increased its interaction with CTX-M-15 and formed a tight structure with the enzyme. Quercetin, which we examined as an inhibitor candidate, increased the number of hydrogen bonds in the cavity to which it was bound and it was concluded that its interaction with the enzyme is continuous. The other two inhibitors, tazobactam, and sulbactam did not change their binding positions. MMGBSA binding energy calculations, which are post-simulation analyses, were calculated and binding affinities were listed as from high to low avibactam > sulbactam > quercetin > tazobactam > clavulanic acid. Quercetin has a similar or better binding affinity than tazobactam, sulbactam, and clavulanic acid inhibitors having a β-lactam structure according to these results. Considering all these results, natural compound quercetin gave similar or better results with tazobactam, sulbactam, and clavulanic acid. Overall, quercetin has been demonstrated in silico studies as a potential inhibitor candidate for CTX-M-15 and has been made ready for in vitro and in vivo studies for further study.

## Ethics approval

Not applicable
